# Forging the iron‐net: Towards a quantitative understanding of microbial communities via siderophore‐mediated interactions

**DOI:** 10.1002/qub2.84

**Published:** 2025-01-10

**Authors:** Shaohua Gu, Jiqi Shao, Ruolin He, Guanyue Xiong, Zeyang Qu, Yuanzhe Shao, Linlong Yu, Di Zhang, Fanhao Wang, Ruichen Xu, Peng Guo, Ningbo Xi, Yinxiang Li, Yanzhao Wu, Zhong Wei, Zhiyuan Li

**Affiliations:** ^1^ Center for Quantitative Biology Academy for Advanced Interdisciplinary Studies Peking University Beijing China; ^2^ Peking‐Tsinghua Center for Life Sciences Academy for Advanced Interdisciplinary Studies Peking University Beijing China; ^3^ School of Life Sciences Shandong University Qingdao China; ^4^ Jiangsu Provincial Key Laboratory for Organic Solid Waste Utilization Jiangsu Collaborative Innovation Center for Solid Organic Waste Resource Utilization National Engineering Research Center for Organic‐based Fertilizers Nanjing Agricultural University Nanjing China; ^5^ School of Physics Peking University Beijing China; ^6^ College of Computer Science and Technology (College of Data Science) Taiyuan University of Technology Taiyuan China

**Keywords:** ecology, evolution, iron‐net, microbial community, siderophores

## Abstract

Iron is a critical yet limited nutrient for microbial growth. To scavenge iron, most microbes produce siderophores—diverse small molecules with high iron affinities. Different siderophores are specifically recognized and uptaken by corresponding recognizers, enabling targeted interventions and intriguing cheater‐producer dynamics. We propose constructing a comprehensive iron interaction network, or “iron‐net”, across the microbial world. Such a network offers the potential for precise manipulation of the microbiota, with conceivable applications in medicine, agriculture, and industry as well as advancing microbial ecology and evolution theories. Previously, our successful construction of an iron‐net in the *Pseudomonas* genus demonstrated the feasibility of coevolution‐inspired digital siderophore‐typing. Enhanced by machine learning techniques and expanding sequencing data, forging such an iron‐net calls for multidisciplinary collaborations and holds significant promise in addressing critical challenges in microbial communities.

## 1 INTRODUCTION

**FIGURE 1:**
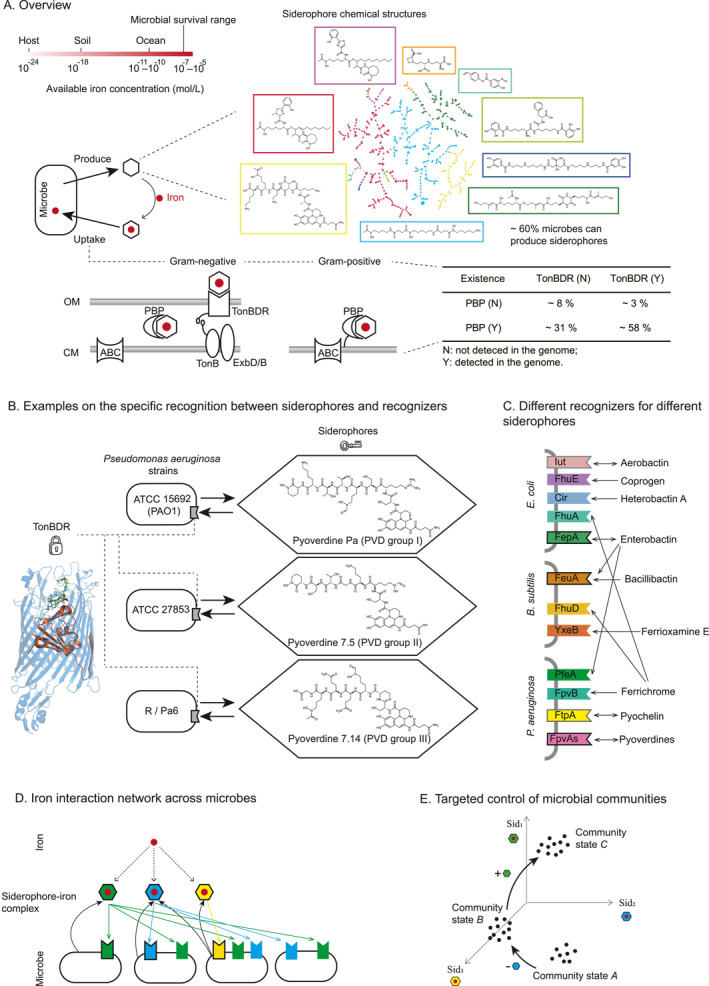
An iron interaction network mediated by diverse siderophores offers insights and controls over microbial communities. (A) Overview of Siderophore‐Microbe Relationships. Ferric iron concentrations in various environments are significantly below microbial requirements (upper left). Microbes produce siderophores to chelate and uptake iron (lower left). The chemical diversity of known siderophores are extracted from in Siderophore Information Database (SIDERITE) [16], shown in the Tree‐MAP (TMAP) view (upper right), and illustrated with examples. Different siderophore uptake mechanisms in Gram‐negative and Gram‐positive bacteria are shown, highlighting the distribution of TonB‐dependent receptors (TonBDRs) and periplasmic binding proteins (PBPs) (lower panel). Each siderophore has a specific TonBDR that facilitates its uptake by the bacterial cells. The energy required for this transport is provided by the TonB–ExbD/B complex. This complex functions as a molecular ‘motor’, which transmits energy from the cell inner membrane (CM) proton motive force to the TonBDRs in the outer membrane (OM), facilitating the translocation of the siderophore–iron complex into the periplasm by a specific ATP‐binding cassette (ABC) transporter. (B) Recognition of pyoverdine siderophores by specific receptors in different *Pseudomonas*. The names of three siderophores can be searched in siderophore information database. Pyoverdine receptors are indicated by notched rectangles. (C) Cross‐utilization of siderophores among different species. Double arrow indicates that this species can produce and update this siderophore, and the corresponding recognizers are marked with edges. Meanwhile, single arrow indicates that this species only can update (but not produce) this siderophore and the corresponding recognizers have no edges. All siderophore names shown in this plot can be searched in the siderophore information database. (D) A scheme of microbial interaction networks medicated by siderophores. Different types of siderophores (indicated by colors) and their corresponding recognizers on microbial membranes are shown. Microbes are represented by rounded rectangles, siderophores by colored hexagons, and recognizers by notched rectangles. Recognizers can uptake siderophores of the same color. Production is indicated by black arrows, and utilization by arrows in the corresponding siderophore colors. The same settings are used for illustrating siderophore interactions in subsequent figures. (E) Schematic of using different siderophores (Sid) to regulate microbial community states.

Ironically, most microbes on Earth are limited by iron [1]. Iron is the most abundant element by mass in the Earth’s crust [2] yet genuinely not bioavailable to microbes. This scarcity has been caused by microbes themselves as cyanobacteria oxygenated the atmosphere through billions of years of photosynthesis [3]. Since the Great Oxygenation Event, the oxidation of iron has led to its precipitation as insoluble iron (III) oxides, significantly reducing the availability of bioavailable iron in most natural habitats [4]. Consequently, the concentration of free iron in these environments is much lower than the levels needed by microbes for optimal growth (Figure 1A, upper left). For example, while bacteria generally require iron concentrations around 10^−7^–10^−5^ mol/L to thrive [1], the available iron in the ocean ranges from 10^−11^–10^−10^ mol/L [5], in soil around 10^−18^ mol/L [6], and in human extracellular environments as low as 10^−24^ mol/L [7]. Although bioavailable iron increases with decreasing pH and oxygen levels, even in low‐pH (as low as 4) its concentration is still below the threshold for microbial growth [8]. In low‐oxygen environments like the gut competition for iron remains intense [9, 10]. Given iron’s essential role in processes like energy metabolism and DNA repair, microbial competition for iron is a key factor shaping microbial communities in most natural habitats [11, 12].

Microbes use several mechanisms to acquire limited iron from their environment including siderophore‐mediated uptake, direct uptake from host proteins (such as heme and transferrin), and reductive iron uptake [13]. Among these, siderophore‐mediated iron uptake is the most dominant [12]. Siderophores are a class of small iron‐chelating molecules with varying iron‐binding affinities, produced and secreted by microbes into the environment [14]. They bind to iron, forming siderophore–iron complexes that are specifically recognized and taken up by microbial cells [13] (Figure 1A, lower left). Compared to the various pathways for acquiring other essential nutrients like carbon and nitrogen, microbial iron uptake is relatively streamlined and concentrated, involving the following two main components: (a) the production of siderophores and (b) their recognition and uptake by the microbial cells.

Despite being essential for microbial survival, siderophores exhibit remarkable structural diversity (Figure 1A, upper right). At least 700 siderophore structures have been experimentally determined [15], and there could be thousands of iron‐binding structures in the vast natural products repository [16]. Moreover, bioinformatic approaches have revealed that structural diversities, even within well‐studied genera like *Pseudomonas*, are largely hidden [17]. Moreover, siderophore is among the largest classes of microbial secondary metabolites [18]. Our preliminary computational analysis, performed through genome annotation using a Poisson hidden Markov model to search microbial genomes for siderophore synthetase and recognizer genes, indicates that around 60% of microbial genomes contain at least one siderophore synthetase pathway. This highlights the vast, yet untapped, potential for siderophore diversity.

The other key components, recognition and uptake, differ significantly between Gram‐negative and Gram‐positive microbes (Figure 1A, lower right) [19]. In Gram‐negative bacteria, siderophore–iron complexes are recognized by TonB‐dependent receptors (TonBDR) and transported across the outer membrane, through the periplasm, and into the cytoplasm via ATP binding cassette (ABC) transporters [20]. Conversely, Gram‐positive bacteria, which lack an outer membrane, use membrane‐bound periplasmic binding proteins (PBP) to recognize the siderophore–iron complexes, then the ABC transporters to directly transport them across their cell wall [21, 22]. Intriguingly, in our preliminary estimation, while 40% of microbes are incapable of producing siderophores, only around 8% lack these siderophore recognition machinery (does not possess TonBDR nor PBP in genome). This suggests interesting ecological interplay, where nonproducing microbes may rely on siderophores produced by others within their community.

The specific recognition relationship between various siderophores and their corresponding recognizers is crucial in microbial iron scavenging. Intriguingly, previous research has shown that different siderophores are preferentially recognized and taken up by specific recognizers [23, 24, 25]. For example, experimental evidence indicates that three different strains of *Pseudomonas aeruginosa* (*P*. *aeruginosa*) each produce three types of pyoverdines (the typical siderophore in *Pseudomonas* genus) and can only utilize the pyoverdine they produce, with minimal cross‐recognition or cross‐uptake (Figure 1B) [24, 25]. Despite the structural similarities among pyoverdine receptors (FpvAs) in these strains, subsequent structural studies identified sites in the loop L7 and the plug domain that influence receptor selectivity [26, 27]. Recent bioinformatic analyses suggested that sequence regions near the plug domain are most strongly associated with pyoverdine selectivity in *Pseudomonas* [17]. The pyoverdines and their matching receptors form specific recognition relationships, much like keys fitting into locks.

Meanwhile, siderophore piracy, where microbes utilize siderophores not produced by themselves, is widespread. This phenomenon occurs not only within phylogenetically close species but also extends across genera, families, and even kingdoms [28, 29, 30]. Studies investigating natural habitats have shown that uncultured strains frequently utilize siderophores produced by nearby species [31]. Other examples come from well‐characterized model organisms. For instance, *Escherichia coli* (*E. coli*) can uptake not only the enterobactin it produces but also aerobactin (produced by *Klebsiella* and some pathegenic *E. coli*), ferrichrome (produced by multiple fungi), coprogen (produced by *Penicillium*) and heterobactin A (produced by *Rhodococcus*) [32, 33] (Figure 1C). Similarly, the Gram‐positive *Bacillus subtilis* (*B. subtilis*) produces bacillibactin and can also uptake enterobactin, ferrichrome, and ferrioxamine *E* (produced by *Streptomycetes*) [34, 35, 36, 37]. *P*. *aeruginosa* produces pyoverdine and pyochelin and can pirate ferrichrome and enterobactin [38, 39, 40], though it cannot utilize the structurally similar bacillibactin [41]. Within the diverse types of pyoverdines produced by the *Pseudomonas* genus most *P*. *aeruginosa* strains are stringent in only uptaking self‐produced pyoverdines [24, 42]. However, other *Pseudomonas* species, such as *Pseudomonas fluorescens* and *Pseudomonas putida*, frequently uptake different pyoverdines produced by other strains [29, 43].

How can extensive cross‐utilization between microbes occur despite the stringent specific recognition between siderophores and their recognizers? The answer lies in the genetic makeup of most microbes, which usually possess multiple recognizers in their genomes [44]. Besides the self‐recognizers for their own siderophores, these additional recognizers can match the lock‐key relationship of various target siderophores, allowing a strain to uptake siderophores from other producers [25, 33, 35, 38, 45] (Figure 1C).

In brief, we envision a comprehensive “iron‐net” (Figure 1D), an iron interaction network mediated by various siderophores across microbes. We believe that such network would provide a sharp cutting point to “poke” into the microbial community. The universal need for iron and the widespread presence of siderophore production and uptake genes ensure the broad applicability of this approach. Meanwhile, the diversity of siderophores, coupled with their specific lock‐key relationships with recognizers, provides room for differentiation. Although depicted ideally in Figure 1D, these relationships are not strictly one‐to‐one; for instance, recognizers in *E. coli* and *B. subtilis* can uptake multiple siderophore types, and a single siderophore, such as enterobactin, can be recognized by different recognizers in Gram‐positive and Gram‐negative bacteria (Figure 1C). Despite this, recognizers generally show strong preferences for specific siderophores. This discriminative power allows for differentiation of microbes in the siderophore chemical space.

The power of this differentiation is significant. For instance, in microbial communities with varied health states, such as different enterotypes [46], adjusting siderophore production may shift the balance toward a healthier state (Figure 1E). Past studies have demonstrated the effectiveness of iron deprivation in suppressing pathogens, by siderophores they cannot utilize [47]. The earliest evidence of it came from a 1980 study conducted by Kloepper and colleagues, who found that soil pathogens were deprived of accessible iron due to the production of iron carriers by a *Pseudomonas* strain isolated from potato peels or roots [48]. Furthermore, it is commonly believed that *B. subtilis*’s anti‐microbial properties stem from its capacity to generate bacillibactin, a siderophore that its competitors do not employ [34, 35, 41]. Conversely, shared siderophores can support the growth of microbes capable of utilizing them [29, 31, 34]. In brief, siderophores act as a double‐edged sword—benefiting microbes that can use them while inhibiting those that cannot by sequestering iron [49]. Overall, the generality, diversity, and specificity of this “iron‐net” present opportunities for targeted interventions under iron limited conditions, potentiating the rational manipulation and design of complex microbiotas.

## 2 FROM SEQUENCE TO ECOLOGY: HARNESS THE MOLECULAR COEVOLUTION TO PREDICT SIDEROPHORE‐MEDIATED INTERACTIONS

The fundamental step to construct such an iron‐net is to perform “siderophore‐typing” for each microbe [24, 50]. This involves identifying which types of siderophores a microbe produces (if any) and which types of siderophores it can uptake. In this context, we use the term “type” to refer to a set of structurally similar siderophores that can be uptaken by a set of highly homologous recognizers.

The primary challenges in siderophore‐typing all microbes are the limited experimental capacities and the bioinformatic complexities. Bioinformatic analyses often identify multiple recognizers within a genome [44], making it challenging to distinguish between “self”‐recognizers (those that uptake the types of siderophores produced by the same strain) and “cheating”‐ recognizers (those that uptake types of siderophores not produced by this strains) [45]. The widespread cross‐interactions between phylogenetically distant species add further complexity. However, the correspondence between genetic sequences and functional specificity, such as recognizers’ specificity and its genetic sequences, and siderophore products with their synthetase pathways, offers potential solutions. By these gene‐function mappings, it is possible to reconstruct the iron‐net from microbial sequences.

We propose that molecular coevolution can be harnessed to identify the lock–key pairs of siderophores and their recognizers. The basis for such coevolution lies in the fact that siderophores are costly secondary metabolites. Take *Pseudomonas* as example, it has been experimentally shown that the growth rate of pyoverdine producers is 3%–10% lower than that of nonproducers [51, 52]. As a result, cells must ensure they can efficiently uptake the siderophores they produce to offset these high production costs.

**FIGURE 2:**
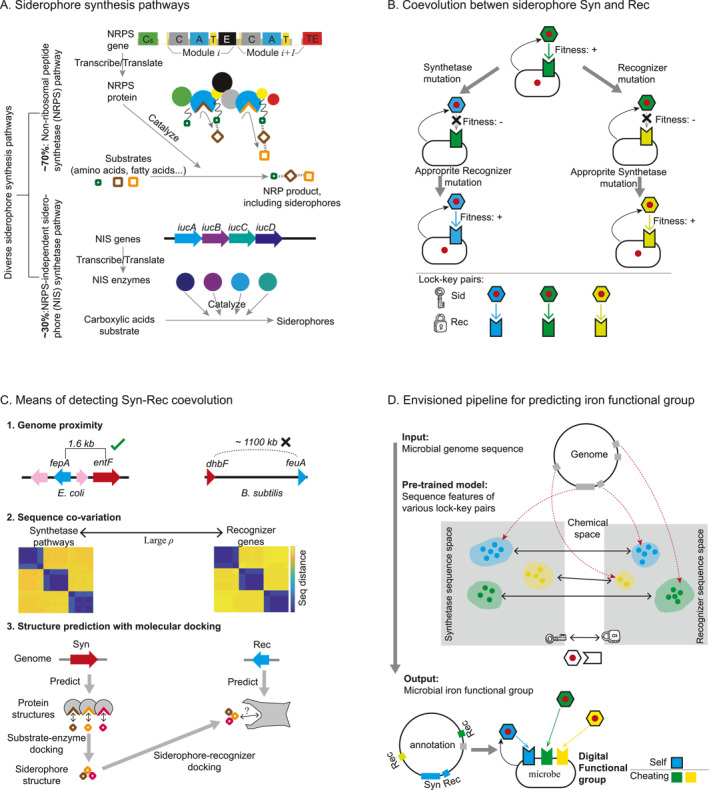
Digital siderophore‐typing based on sequences. (A) Overview of the two main classes of siderophore synthesis pathways: nonribosomal peptide synthetase (NRPS) (upper) and NRPS‐independent siderophore (NIS) (lower). Approximately 70% of microbial siderophores are synthesized by NRPS, while about 30% are synthesized by NIS. Cs (Starter condensation domain); C (Condensation domain); A (Adenylation domain); T (Thiolation domain); E (Epimerization domain); and TE (Thioesterase domain). (B) Illustration of the selection pressure driving structural coevolution between siderophores and their recognizers. This plot uses the same settings as Figure 1D for illustrating siderophore interactions. Syn (Syntheatse); Rec (Receptor). (C) Three computational methods for detecting coevolution between siderophore synthetases and recognizers. (D) Proposed pipeline for predicting siderophore functional groups from microbial genomes. The functional group is characterized by a two‐element vector identifying the types of siderophores produced and utilized by each strain. Self (Self‐receptor) and Cheating (Cheating‐receptor).

Microbes generally use two main pathways for siderophore production [14]: the nonribosomal peptide synthetase (NRPS) pathway and the NRPS‐independent siderophore (NIS) synthetase pathway (Figure 2A). NRPS pathways are utilized more frequently than the NIS pathway, responsible for around 70% of structurally known siderophores [16]. The NRPS pathway operates in an assembly line manner, where each module selects and adds specific components to the growing peptide chain, resulting in a diverse array of siderophores [18]. Of note, each module is approximately 1,000–1,300 amino acids in length, and a siderophore NRPS pathway typically contains three to eight modules, making this gigantic enzyme expensive to synthesize. The NIS pathways are shorter in nucleotide length in general yet still involve multiple enzymes that catalyze the synthesis of siderophores from carboxylic acid substrates [53, 54]. Besides enzyme synthesis, both NRPS and NIS pathways may compete with protein synthesis for the amino acid pool imposing further metabolic costs on the cells. Experimental evidence have suggested that forced production of siderophores when iron is nonlimiting decreases the fitness of microbes [47], supporting the high cost of synthesizing siderophores.

Given the cost of synthesis, structural coevolution between siderophores and their corresponding recognizers is reinforced by natural selection [11]. A microbe with matching siderophores and recognizers can efficiently uptake the necessary iron, maintaining its fitness (Figure 2B, top panel, with both siderophore and recognizers shown in green). However, mutations in the siderophore synthetase (left arrow, resulting in new type of siderophores illustrated in blue) or in the recognizers (right arrow, changing recognizers into a new yellow type) lead to mismatches. Unmatched siderophores and recognizers fail to transport iron into the cell, causing the microbe to incur the costs of siderophore synthesis without reaping the benefits, thereby reducing its fitness. Appropriate mutations that either revert the changes in siderophore or recognizer structures back to the original type or further mutate the sequences to create new matching lock–key pairs can restore the iron‐scavenging machinery. This ensures that the microbe’s investment in siderophore production is compensated (Figure 2B, bottom panel). Under the assumption that “cells need to be able to uptake the siderophores they produce”, various lock–key pairs of siderophores and recognizers evolve, with concurrent changes in their key sites demonstrating coevolution.

Three sequence‐based computational approaches can help establish the lock–key relationships between siderophores and their recognizers. The first is by genome approximity (Figure 2C, first panel). In many microbes, siderophore synthetase genes are located close to their corresponding recognizer genes, facilitating coevolution through genome rearrangements [55]. For instance, in *E. coli*, the enterobactin synthetase and its receptor FepA are within the same biosynthetic gene cluster [56]. In *Pseudomonas*, the majorities of self‐recognizers lie within 10 kb of the synthetase [57]. However, this proximity rule has exceptions, such as in *B. subtilis*, where bacillibactin synthetase is over 1,000 kb away from the periplasmic binding protein FeuA [58].

Recently, our work exploited the sequence co‐variation between synthetase and recognizer genes to reconstruct the pyoverdine‐mediated interaction network in *Pseudomonas* [57] (Figure 2C, second panel). The approach assumes that the strongest coevolution signals occur between matching synthetase and recognizer pairs. Algorithms maximizing coevolution strength can identify these “self‐recognizer” from the many possible recognizer in the genome. This method is not limited by genome co‐localization of the iron‐scavenging machineries yet requires a large number of sequences for accurate detection of coevolution signals. It has shown high prediction accuracy in experimental validations.

Leveraging advances in large models of structure prediction and molecule docking [59, 60], a third approach can be developed in the near future to predict interactions at a molecular level (Figure 2C, last panel). Two levels of protein–ligand dockings are required. First, the structure of the synthetase responsible for siderophore biosynthesis can be inferred from genomic sequences, enabling the prediction of the small molecule siderophore’s structure. Significant progress has been made in predicting nonribosomal peptide synthetase (NRPS) products [18, 61], and our recent algorithm specifically tailored for *Pseudomonas* pyoverdine has achieve accuracy rate of 95% [17]. Predicting NIS product has also been advancing [54, 62, 63]. The growing dataset of known siderophore structures can serve as a valuable training set for further refinement of these models [16].

Secondly, the structure of siderophore receptors such as TonB‐dependent receptors (TonBDR) or periplasmic binding proteins (PBP) can be predicted from their sequences. By docking these receptors with siderophores, it is feasible to mechanistically determine their lock‐and‐key relationships [60]. Although fewer than 20 structures of TonB‐dependent receptors and membrane‐bound PBPs have been experimentally characterized, significant progress has been made in sequence‐to‐structure prediction in recent years [20, 59]. Additionally, molecular dynamics simulations have shown promising results in understanding the selectivity of TonBDRs for various siderophores [64, 65, 66]. Furthermore, the lock‐and‐key relationships identified through these approaches can further serve as training sets for siderophore‐receptor docking algorithms, helping to expand and refine these models.

Current rapid expansion of microbial sequence data offers new opportunities to understand the iron interaction behaviors of microbes [67]. We envision a pipeline to predict these behaviors from any sequenced microbial genome (Figure 2D). The input would be the genome sequence, processed by a pretrained model that has learned lock–key relationships from extensive microbial sequence data, using the three approaches or a combination thereof. A prototype of such pretrained model has been realized in the *Pseudomonas* genus, where the lock–key relationship learnt from the whole genus were used to predict the iron interaction networks within each species. This pretrained model should connect lock–key pairs in two sequence spaces: synthetases producing similar siderophores and recognizers selecting these siderophores. Although interactions occur in the chemical space, we assume that sequence similarity correlates with functional similarity. Thus, any query gene sufficiently similar to one type of lock–key pair in the sequence space would behave as a member of this type.

The output is the “microbial iron functional group”, indicating which types of siderophores the microbe produces and uptakes. By this output, recognizers in a microbe can be classified into self‐recognizers (uptaking its own siderophores) and cheating recognizers (uptaking siderophores from other organisms). Mathematically, it can be represented by a two‐element digital vector, characterizing the microbe’s iron acquisition and competition strategies. If such “from sequence to functional group” digital siderophore‐typing can be realized in general, the expanding sequence data will enable researchers to investigate iron interaction networks across the microbial world, opening up intriguing explorations into ecological and evolutionary dynamics.

## 3 THE IRON INTERACTIONS NETWORK AS A PLAYGROUND FOR MICROBIAL GAME THEORIES

**FIGURE 3:**
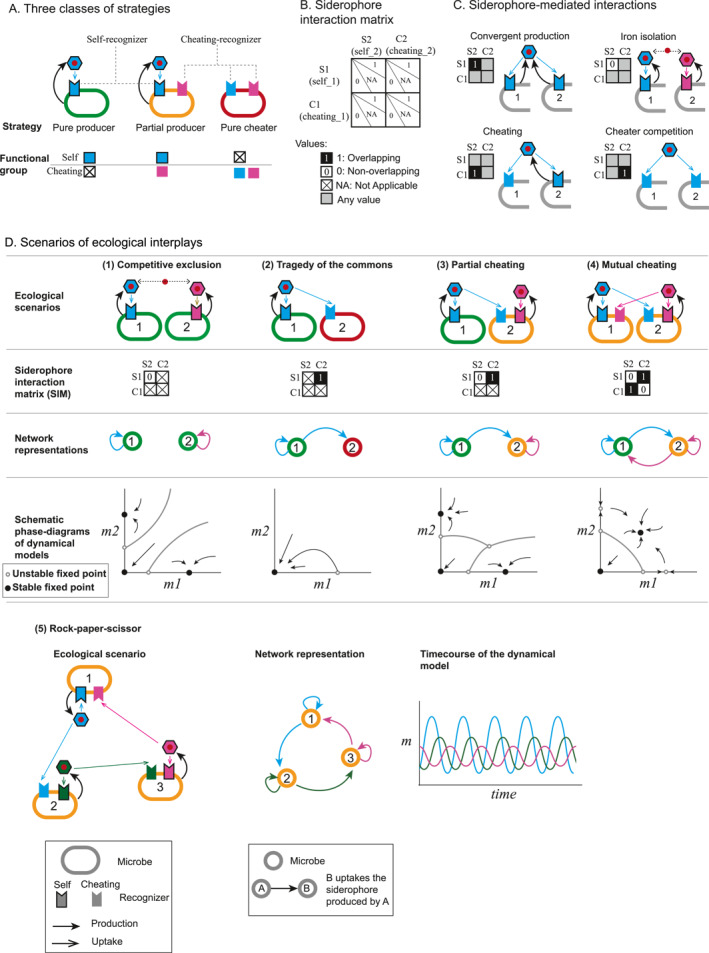
Siderophore‐mediated interactions can manifest diverse ecological interplays. (A) Three classes of strategies for iron acquisition are differentiated by each microbe’s siderophore production and uptake behaviors with their functional groups illustrated in the bottom panel. Self‐recognizers are marked with edges, and cheating‐recognizers have no edges. A box with a cross indicates “nonexistence”. All plots in this figure use the same settings as Figure 1D for siderophore‐mediated interactions, with the edge colors of microbes indicating the classes of strategies (Green: pure producer; orange: partial producer; red: pure cheater). (B) The siderophore‐interaction matrix (SIM) characterizes the interactions mediated by siderophores between two microbial strains. Each element in the matrix indicates whether the self‐ and cheating‐recognizers of the two strains overlap in type (denoted by 1 and 0 values) or if such recognizers are absent (denoted by an ‘NA’ value). ‘Any value’ indicates that overlap or non‐overlap of recognizers is acceptable. (C) Demonstration of the interactions characterized by different elements in the SIM matrix, including convergent production (upper left, by SIM (1,1) = 1), iron isolation (upper right, by SIM (1,1) = 0), cheating (lower left, by SIM (1,2) = 1 or SIM (2,1) = 1), and cheater competition (lower right, by SIM (2,2) = 1). (D) Classical scenarios of ecological interplays that can be realized by siderophore‐mediated interactions such as competitive exclusion, the tragedy of the commons, partial cheating, and mutual cheating (from left column to right column, D (1) to D (4)). Each column shows, from top to bottom, the strains’ production and utilization relationships, the corresponding SIM, the simplified network representation using arrows from strain *i* to *j* to indicate that the siderophore made by strain *i* can be utilized by strain *j*, and the schematic phase‐diagrams of the dynamic model. Additionally, a three‐strain system has the potential to exhibit a ‘rock‐paper‐scissors’ dynamic, which is also shown in D (5).

Iron interactions among microorganisms create complex and fascinating ecological dynamics. Iron, being the only limiting nutrient in this game, is divided into various “sectors” by different types of siderophores [68]. For each type of siderophore–iron complexes, microbes possessing the matching recognizers uptake them as public goods, whereas the others see them as “public bads” that prevent them from accessing iron [49]. From the perspective of production and cheating, multiple iron strategies can emerge. In *Pseudomonas*, we observed the following three classes of iron acquisition strategies [57] (Figure 3A): 1. “Pure producers” that produce siderophores and only uptake the classes of siderophores they produce, lacking cheating recognizers. 2. “Partial producers” that produce/uptake their own siderophores and also absorb siderophores produced by others, possessing both self‐ and cheating‐recognizers. 3. “Pure cheaters” that do not produce siderophores while uptaking siderophores produced by others, possessing only cheating recognizers. Different habitats and lifestyles exhibit distinct preferences for iron strategies. Notably, various pathogenic lifestyles consistently correlate with the lack of partial producers in the community, raising further curiosity about the ecological interactions mediated by these iron acquisition strategies [57].

We can utilize a siderophore interaction matrix (SIM) to analyze the complex interplays in siderophore‐mediated iron interactions between two microbes (Figure 3B). Each strain’s iron functional group is characterized by a two‐element vector identifying its self and cheating recognizers. This creates a 2 × 2 matrix describing the overlaps between two strains’ self and cheating recognizers. Each element of this matrix represents different types of interactions.

For example, if the self‐recognizers of two microbes belong to the same type, the $\text{sel}f_{1}$–$\text{sel}f_{2}$ element of the matrix will be nonzero, indicating the convergent production of the same siderophores (Figure 3C, upper left plot). Conversely, if the $\text{sel}f_{1}$–$\text{sel}f_{2}$ element is zero, it indicates that the two microbes produce different siderophores, leading to direct iron competition (Figure 3C, upper right plot). When the self‐recognizer of microbe two overlaps with the cheating‐recognizer of microbe 1, cheating occurs, where microbe two exploits the siderophore production of microbe 1 (Figure 3C, lower left plot). When both microbes share the same cheating recognizers (Figure 3C, lower right plot) they can compete to exploit another microbe. Multiple interactions can occur simultaneously between two microbes, illustrating the complexity of microbial iron interactions.

Many classical scenarios in microbial game theory can be realized through different combinations of self‐ and cheating‐recognizers in microbiota, alongside intriguing new games that warrant further interdisciplinary investigations (Figure 3D). Classical competitive exclusion [69], or “winner takes all”, is demonstrated by microbes producing different siderophores and engaging in direct iron competition (Figure 3D(1)). Without cross‐utilization, the strain that isolates iron more effectively or has a higher initial inoculation wins, eliminating the others. In general, producing siderophores that cannot be utilized by competitors has been considered as an effective competitive strategy [70], as seen with *B. subtilis*, which antagonizes *Pseudomonas* using its siderophore bacillibactin [37]. Experimentally, two strains of *P*. *aeruginosa* producing different pyoverdines have been shown to be unable to coexist in well‐mixed cultures, demonstrating competitive exclusion [71].

The “tragedy of the commons” [72] occurs when a producer is exploited by a pure cheater strain, leading to the extinction of both microbes (Figure 3D(2)). Experimentally, cheater strains of *P. aeruginosa* that do not secrete pyoverdine can outcompete the producer strain under various conditions, causing the collapse of cooperation [47] or reduced fitness [73]. Addressing this dilemma of cheating and cooperation has been an area of intense study, with various experimental and computational mechanisms proposed to maintain cooperation in the presence of cheaters [52, 71, 74, 75, 76].

Nevertheless, the quantitative characterization of ecological interactions mediated by diverse types of siderophores remains relatively unexplored. Some intriguing phenomena predicted by dynamic models are still awaiting more accurate experimental validation. For example, dynamic models predict that when two microbes produce different siderophores with one cheating on the other, the scenario remains “winner takes all”, but the partial producer (microbe 2) gains an advantage over the pure producer (microbe 1). This makes microbe 2 more resistant to invasion by microbe 1 and microbe 1 more capable of invading microbe 2 (Figure 3D(3)). This biased advantage toward partial cheaters can be utilized for pathogen control [57, 77].Interestingly, when both strains cheat on each other while producing their own siderophores, mutual piracy allows coexistence under appropriate cheating strength [76] (Figure 3D(4)). This opens intriguing discussions on how cheating may facilitate biodiversity. Also, the “rock–paper–scissors” dynamic, a nonlinear phenomenon with significant implications for dynamic coexistence, raises researchers’ interest in both synthetic and natural systems [78, 79, 80]. Although it has been challenging to find microbes with such cyclic relationships in nature [79, 81], we propose that this dynamic might be easily realized in siderophore‐mediated interactions. Three partial producers, each producing a siderophore type that can be utilized by the following strain, could exhibit this relationship (Figure 3D(5)).

Taken together, the intricate iron interactions among microbes create an exciting playground for microbial game theory, offering profound insights into ecological dynamics and biodiversity. This framework sets the stage for further interdisciplinary research and innovative approaches to understand the design principle and to manipulate microbial communities.

## 4 FIGHTING FOR DIVERSITY: THE ECO‐EVOLUTIONARY IMPLICATIONS OF MICROBIAL CHEMICAL INNOVATIONS VIA SECONDARY METABOLISM

High diversity of siderophore types is crucial for the complex ecological interactions described. Under intense selection pressure for iron acquisition, how and why have so many different siderophores evolved instead of converging on the most effective siderophore? What are the ecological and evolutionary implications of this diversity?

**FIGURE 4:**
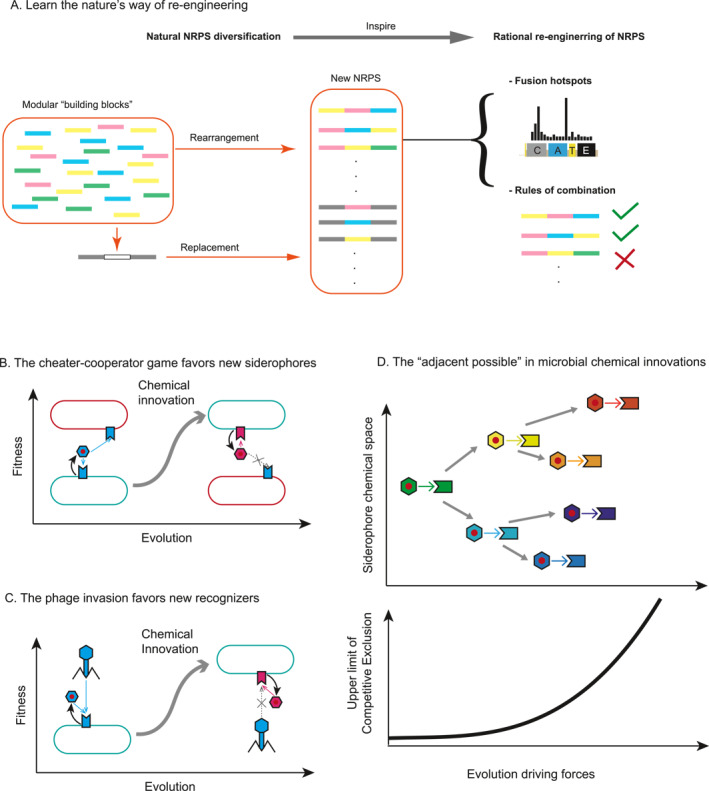
Evolutionary and molecular basis, and implications of siderophore diversification. (A) Illustration of how siderophore diversification can help us understand nature’s method of reengineering. Natural NRPS diversification, such as the rearrangement and replacement of modular building blocks, inspires rational reengineering of NRPS to create new products by understanding fusion hotspots and combination rules. (B–C) Illustration of how the cheater‐cooperator game between microbes (B) and the phage selection (C) favors the creation of new siderophore lock–key pairs. Plots use the same settings as Figure 1D for siderophore‐mediated interactions. (D) The concept of “adjacent possible” in microbial chemical innovations. The cartoon in the upper panel shows how an existing siderophore lock–key pair (green type) expand into its adjacent possibilities (different colors) due to certain evolutionary driving forces. The lower panel demonstrates how this diversification of siderophores increases the upper limit of competitive exclusion.

At the molecular level, the repetitive modular structure and assembly line working style of NRPS provide an excellent platform for evolving chemical diversity (Figure 4A) [18, 82, 83]. Rearrangement within this modular enzyme system is enabled by conserved motifs in each module, while mutations in adenylation (A) domains alter substrate selectivity [84, 85]. These changes synergistically contribute to the amazing diversity of nonribosomal peptides (NRPs). Insertion, deletion, or replacement of large DNA fragments drive backbone alterations, while point mutations fine‐tune substrate specificity. This high diversity fuels microbial arms races, explaining why NRPS pathways are among the most abundant secondary metabolism pathways in microbes utilized for numerous competitive agents including siderophores and antibiotics [82, 86, 87, 88]. Conversely, the less common NIS pathway for siderophore synthesis remains less explored in terms of chemical diversity and underlying mechanisms.

The practical implications of this molecular diversification extend to the rational reengineering of NRPSs. Inspired by the pharmaceutical potential of natural products and the “lego‐like” assembly of modular enzymes, researchers have been attempting to manipulate NRPS enzymes to create novel products [89]. However, piecing together NRPS modules from different sources has proven notoriously difficult, with directed mutations in substrate‐specifying regions often resulting in dysfunctional enzymes [90]. In contrast, natural processes have continually reengineered NRPSs to produce diverse siderophores. For instance, nearly 200 pyoverdines have been predicted in the *Pseudomonas* genus, with some differing by only one or two modules, suggesting they are connected by single recombination events [57]. These numerous evolutionary events that shape diverse siderophores provide a wealth of information on natural fusion hotspots and the rules of combination that produce viable products (Figure 4A) [91]. By studying these natural processes, researchers can gain valuable insights to guide the engineering of NRPSs [92]. Nature remains a vital teacher in the quest for innovation.

At the level of driving forces, a longstanding question is whether and why generating new siderophores confers evolutionary benefits. The simplest possibility is no—neutral drift can generate new types of siderophores and recognizers without involving selections [93]. However, the relative conservation of other resource uptake pathways suggests an exceptional diversification rate in the iron‐scavenging system [94]. Positive selection pressure among FpvA receptors in the *Pseudomonas* genus further indicates that neutral drift alone may not be sufficient to explain this phenomenon [95].

One explanation involving selection is that the diversification of siderophores provides an advantage by helping producers evade cheaters [95, 96] (Figure 4B). Even small changes in the siderophore structure can substantially alter its recognizer specificity, escaping the piracy from other competitors [97]. Given the prevalence of piracy in the siderophore world this is a plausible explanation [11]. Nevertheless, some highly conserved siderophores such as enterobactin and ferrichromes [30, 40, 98, 99] are frequently exploited by cheaters without evolving into new forms, making researchers wonder their mechanisms of resisting cheating.

Another possible explanation is that it is the recognizers, rather than the siderophores themselves, that need to change due to phage selection (Figure 4C) [95]. Phages can utilize the iron‐scavenging pathway to enter microbes [100, 101, 102]. *E. coli* has been shown to produce siderophore and even other metabolites to occupy the TONB‐dependent receptors to prevent them from being utilized by phage [103, 104]. Under such selection pressure, recognizers can mutate to escape phages which then subsequently lead to changes in siderophore structures. Although multiple theories all provide some compelling narrative, no definitive answer has yet been achieved due to lack of a global overview on the diversity of siderophores.

Additionally, as many siderophores are considered virulence factors for various pathogens and are crucial for some to colonize their hosts [49], the host‐microbe interactions can impose positive selection pressures on siderophores [7, 11]. Host immune systems have evolved ways to eliminate iron from pathogens, like that neutrophils express lipocalin‐2 to sequester enterobactin [105]. Conversely, enterobacteria evade this sequestration by producing glucosylated derivatives of enterobactin [106]. This ongoing tug‐of‐war between host and bacteria exerts selection pressures on microbial siderophores to adapt and change.

Irrespective of the potential driving forces, the established fact that siderophores exhibit significant diversity has substantial implications for microbial chemical innovations. “Chemical innovation” is a comprehensive term describing how life generates molecules not yet present in its environment, including new siderophores, novel antibiotics, and any other new metabolites [107]. Stuart Kauffman, in his book “Reinventing the Sacred: A New View of Science, Reason, and Religion”, discussed the idea that innovation occurs when life explores the immediate possibilities just beyond the current boundaries of what is known or possible, which expands the “sampling space” of life and fosters diversity [108, 109, 110]. This concept is demonstrated by the way microbes shape their own niches, not only consuming existing resources but also keeping contributing new chemicals [68]. The emergence of new metabolites exponentially expands the chemical space, mediating new interactions that may drive phase transitions in the community [76]. With increased chemical dimensions, competitive principles may no longer limit biodiversity, as life itself pushes up the boundaries (Figure 4D). Investigating the driving forces and subsequent consequences of chemical innovation, both within and beyond the realm of siderophores, can provide profound insights into the self‐organization principles of the microbial world and beyond.

## 5 DISCUSSION

From a practical perspective, the envisioned iron‐net has the potential to provide targeted intervention in microbial communities. Microbes and microbial communities are crucial to human health, agriculture, and industry yet we lack precise methods to manipulate microbiota due to their astonishing complexity. Indeed, iron interaction is just one of the many ways microbes interact with each other. Competition for primary nutrients like carbon and nitrogen, antibiotic and toxin wars, and prey–predator interactions between microbes and phages are all significant factors shaping microbial communities [111, 112, 113]. Nevertheless, the critical importance of iron’s catalytic functions in respiration and DNA replication makes iron acquisition an essential condition for the growth of most microbes [114, 115], on top of these various interactions [116]. Additionally, the diverse lock–key pairs in the siderophore world enable targeted interventions, and digital siderophore‐typing provides a feasible method to infer these iron interactions from ever‐expanding sequence data. Currently, there are already reports of microbiota interventions through siderophore‐mediated interactions [10, 117]. In the age of data and machine learning, constructing a comprehensive iron‐net becomes possible and will provide us with means for precise manipulation in this invisible world.

From a theoretical perspective, secondary metabolism may introduce “new ecology” by actively expanding the possibility space for survival. Nearly all life forms create new survival strategies, from microbial antibiotic arms races to human innovations. Traditional ecological theories are largely based on predefined dimensions, with interaction methods already set in the models. The concept of the “adjacent possible” remains mostly theoretical, with limited development in concrete mathematical models. However, investigating how life expands its possibility space could offer novel insights into ecological theory, particularly when considering microbial secondary metabolism. An exciting next step would be to explore the rate of diversification in siderophores either through genome analysis or directed evolution experiments. This would help determine whether the “adjacent possible” framework applies to microbial diversity, especially in better‐studied taxa like *Pseudomonas*.

Despite the practical and theoretical potential of the iron‐net approach, there are important complexities that extend beyond its current framework. First, a significant limitation of the sequence‐to‐ecology approach is that it does not account for the regulatory aspects of siderophore production and recognition. We operate under the simplified assumption that the presence of synthetase or recognizer genes in the genome implies that an organism can produce or utilize the corresponding siderophores. However, in reality, siderophore production and utilization are tightly regulated, influenced by factors such as iron availability, population density, and the presence of competitors [118, 119]. Second, iron‐mediated interactions are linked to broader metabolic processes. For instance, different nutrient limitations can affect the cost of siderophore production [73] and complex regulatory strategies may arises from evolution [120]. Understanding these regulatory mechanisms and how iron metabolism connects to primary metabolism will require a combination of experiments and theories, making it an essential next step in advancing the iron‐net concept.

Still, constructing and understanding the iron‐net itself is still a long journey. Beyond the current progress in the *Pseudomonas* pyoverdine iron‐net, many puzzles remain. For example, why are different siderophores so varied, that some siderophores act as universal currencies in the microbial world, while others are privatized? And can we obtain a systematic overview on siderophore preference in the microbial world? How specific is the siderophore‐recognizer lock–key relationship across the microbial world? Why hasn’t a super recognizer that uptakes all siderophores evolved, and why not microbes all produce enterobactin, the siderophore with the highest iron‐binding affinity? How do cells regulate their iron strategies in response to different environments and competitors? Can we develop a general algorithm for constructing the iron‐net, or do we need to build it genus by genus? How do Gram‐positive and Gram‐negative microbes cross‐talk? Forging the iron‐net is a vast project that requires interdisciplinary collaborations, spanning molecular structures, bioinformatics, ecological modeling, and medical and agricultural experiments. We call for researchers from all related fields who are interested to join this endeavor.

## AUTHOR CONTRIBUTIONS

**Shaohua Gu**: Data curation; writing ‐ review and editing. **Jiqi Shao**: Data curation; writing ‐ review and editing. **Ruolin He**: Data curation; writing ‐ review and editing. **Guanyue Xiong**: Data curation; visualization; writing ‐ review and editing. **Zeyang Qu**: Data curation; writing ‐ review and editing. **Yuanzhe Shao**: Data curation; visualization; writing ‐ review and editing. **Linlong Yu**: Data curation. **Di Zhang**: Data curation. **Fanhao Wang**: Data curation. **Ruichen Xu**: Data curation. **Peng Guo**: Data curation. **Ningbo Xi**: Data curation. **Yinxiang Li**: Data curation. **Yanzhao Wu**: Data curation. **Zhong Wei**: Writing ‐ review and editing. **Zhiyuan Li**: Conceptualization; funding acquisition; visualization; writing ‐ original draft.

## CONFLICT OF INTEREST STATEMENT

All authors of this manuscript declare that they have no conflicts of interest or financial conflicts to disclose.

## ETHICS STATEMENT

This article does not contain any studies with human or animal subjects performed by any of the authors.

## Data Availability

This is a perspective article and does not contain original research data.
